# Organization-wide adoption of computerized provider order entry systems: a study based on diffusion of innovations theory

**DOI:** 10.1186/1472-6947-9-52

**Published:** 2009-12-31

**Authors:** Bahlol Rahimi, Toomas Timpka, Vivian Vimarlund, Srinivas Uppugunduri, Mikael Svensson

**Affiliations:** 1 Department of Medical and Health Sciences, Linköping University, Linköping, Sweden; 2 Department of Computer and Information Sciences, Linköping University, Linköping, Sweden; 3 Department of Social Medicine, Urmia University of Medical Sciences, Urmia, Iran; 4 Department of Health Information Technology, Urmia University of Medical Sciences, Urmia, Iran; 5 Department of Clinical and Experimental Medicine, Clinical Chemistry, Linköping University, Linköping, Sweden; 6 Drug and Therapeutics Committee, Östergötland County Council, Linköping, Sweden

## Abstract

**Background:**

Computerized provider order entry (CPOE) systems have been introduced to reduce medication errors, increase safety, improve work-flow efficiency, and increase medical service quality at the moment of prescription. Making the impact of CPOE systems more observable may facilitate their adoption by users. We set out to examine factors associated with the adoption of a CPOE system for inter-organizational and intra-organizational care.

**Methods:**

The diffusion of innovation theory was used to understand physicians' and nurses' attitudes and thoughts about implementation and use of the CPOE system. Two online survey questionnaires were distributed to all physicians and nurses using a CPOE system in county-wide healthcare organizations. The number of complete questionnaires analyzed was 134 from 200 nurses (67.0%) and 176 from 741 physicians (23.8%). Data were analyzed using descriptive-analytical statistical methods.

**Results:**

More nurses (56.7%) than physicians (31.3%) stated that the CPOE system introduction had worked well in their clinical setting (P < 0.001). Similarly, more physicians (73.9%) than nurses (50.7%) reported that they found the system not adapted to their specific professional practice (*P *= < 0.001). Also more physicians (25.0%) than nurses (13.4%) stated that they did want to return to the previous system (*P *= 0.041). We found that in particular the received relative advantages of the CPOE system were estimated to be significantly (*P *< 0.001) higher among nurses (39.6%) than physicians (16.5%). However, physicians' agreements with the compatibility of the CPOE and with its complexity were significantly higher than the nurses (*P *< 0.001).

**Conclusions:**

Qualifications for CPOE adoption as defined by three attributes of diffusion of innovation theory were not satisfied in the study setting. CPOE systems are introduced as a response to the present limitations in paper-based systems. In consequence, user expectations are often high on their relative advantages as well as on a low level of complexity. Building CPOE systems therefore requires designs that can provide rather important additional advantages, e.g. by preventing prescription errors and ultimately improving patient safety and safety of clinical work. The decision-making process leading to the implementation and use of CPOE systems in healthcare therefore has to be improved. As any change in health service settings usually faces resistance, we emphasize that CPOE system designers and healthcare decision-makers should continually collect users' feedback about the systems, while not forgetting that it also is necessary to inform the users about the potential benefits involved.

## Background

Computerized provider order entry (CPOE) systems are widely referred to a variety of computer-based systems that share common features of automating the clinical ordering process to ensure standardized, legible, and complete orders [1]. The systems provide an opportunity to reduce errors and thereby improve patient safety [2-4]. In general, CPOE systems have helped healthcare organizations and providers to increase safety, reduce errors, improve work-flow efficiency, and increase quality by obtaining relevant patient information and clinical knowledge at the moment of ordering medications [4,5]. These systems may also affect outcomes such as medication and process costs [4,6]. Nevertheless, the results of several studies indicate that CPOE system implementation and maintenance may also have unintended consequences [7,8].

Many studies related to CPOE systems address the quality of care and services and, in particular, adverse events attributable to medication errors [3,9,10]. The increased use of CPOE systems has been reported to enhance legibility, result in faster transmission of orders, support the user's decision-making processes, and reduce errors [11-14]. However, although the benefits of CPOE systems are widely recognized, few healthcare settings have implemented these systems successfully [15]. Based on the fact that the use of a CPOE system involves individuals and depends on organizational context, any organizational plan to implement such a system could be expected to have procedures for collecting and attending to users' opinions. In such efforts, it is important to collect and evaluate users' feedback about the system. Also, previous studies [9,16] have recommended that additional research to make the impact of CPOE systems more observable may improve adoption by users.

In this study, we set out to examine factors that may influence CPOE adoption among physicians and nurses in a large healthcare organization. The diffusion of innovation theory was used to understand physicians' and nurses' attitudes and thoughts about implementation and use of the CPOE system.

### Theoretical Background

Diffusion has been defined by Everett Rogers as "the process by which an innovation is communicated through certain channels over time among the members of a social system," and an innovation is defined as "an idea, practice, or objective perceived as new by an individual, a group, or an organization"[17]. His diffusion of innovation theory outlines five attributes that are important in assessing the diffusion potential of an innovation: relative advantage (is the innovation "better" than the idea it replaced?); compatibility (is it consistent with existing values and needs of users?); complexity (is it hard to understand and use?); trialability (can you experiment with it?); and observability (are results visible to others?). While adoption of any innovation inevitably generates consequences, such consequences can be desirable or undesirable and anticipated or unanticipated [18].

According to Rogers, it is the unintended consequences that are the least studied in an innovation diffusion process. Undesirable, unintended, and unanticipated consequences consist of the adverse events or constraints that have not previously been seen and that have consequences for the effectiveness and efficiency of the system. Once an innovation has been adopted, consequences such as increased effectiveness and efficiency hopefully follow. However, according to Rogers, the consequences of adoption are the least studied aspect of the innovation diffusion process [17].

Several studies have applied diffusion of innovation theory to study the diffusion and adoption of different kinds of health information systems [7,18-20]. For example, Ford et al. [19] found that developing a CPOE system that is user-friendly and easily integrated into hospitals' legacy systems is more likely to achieve widespread adoption. There are only a few studies of unintended consequences related to the implementation of CPOE systems. One exception is the study by Ash et al [18], reporting errors and security concerns, as well as issues related to alerts, workflow, ergonomics, and interpersonal relationships. The authors also conclude that the diffusion of innovation theory framework is a useful tool for analyzing consequences of implementing complex clinical systems.

### Study Context

The study was performed in Östergötland County (population 423,510) in Sweden, where tax-financed healthcare services are provided to the residents by the county council. Computer-based patient record systems have been used at primary health care centers and hospitals in the county for more than 10 years. The county council also supplied other types of computer systems to healthcare providers, such as appointment systems, physician-secretary communication systems for dictation, and an electronic prescribing system. However, these systems were not connected to one another to allow the sharing of information and other functions.

Implementation of a new integrated computerized patient record system was initiated in 2007 as a pilot project at a primary health care center in the western district (Motala) of the county. The implementation process continued from the western to the eastern district (Norrköping), and was finished in the central district (Linköping) by the end of 2008. This new integrated system, developed commercially, provides a comprehensive overview of the patient's health conditions and care. It makes available an infrastructure for sharing patient data between all healthcare care providers within the county council. One component of the integrated system is the CPOE, which supplies information about patients' medications and prescription support functions, and is used to send electronic prescriptions. Previously, an electronic prescribing system was available only for the primary healthcare centers. Currently, the integrated system provides all units with CPOE system functions. The CPOE system was introduced in a step-wise manner throughout the entire county council.

**Figure 1:**
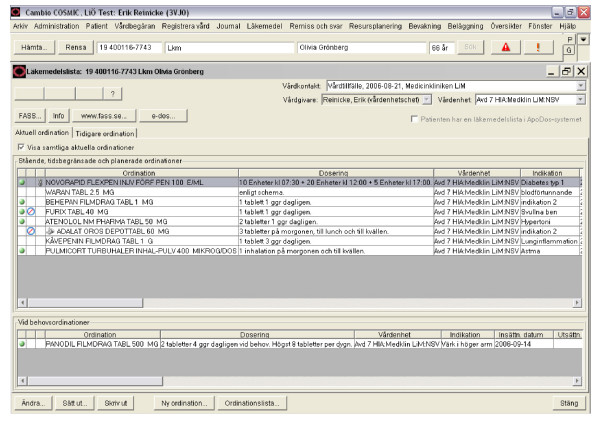
**The physicians' interface to the CPOE system**. A figure showing the physicians' view of the CPOE system.

The CPOE system is built up around a common list of medications comprising current and previous prescriptions. When a prescriber prescribes medication or changes dosage, he or she is supported by a central register of medications that is continually updated, with direct reference to national lists of pharmaceutical specialties, brief descriptions of products, instructions issued with medicines, warnings, and recommended and non-recommended medication and prescription templates [21]. A screen shot of the system interface is shown in Figure 1.

The CPOE used was not a decision support system, i.e. the user was not provided with various alternatives to guide them in their decision making process. It was, however, possible to access concise information from the regional drugs and therapeutic committee regarding recommended drugs and recommended diluents etc.

The introduction of the CPOE system was mandatory for all clinics belonging to the county council. Exceptions were made for clinics where the CPOE did not provide adequate functionality and there was a risk for patient safety, e.g. intensive care clinics. Clinics had to formally apply for permission from a committee convened by the county council. Nurses used the COPE to document the administration of various drugs based on physicians' orders. Nurses had to document if they had prepared and/or administered drugs as per physicians' orders. The functionality for administering infusions was not deemed adequate by the coordinating committee and a number of clinics, for example surgery where large quantities of fluids are administered on a daily basis, were allowed to document their infusions on a standardized paper-based dosage form.

## Methods

### Survey Questionnaire

Two online survey questionnaires based on diffusion of innovation theory were developed to capture data from physicians and nurses, respectively (see Additional files 1 and 2). Three diffusion of innovation theory attributes (relative advantages, complexity, and compatibility) that are necessary for assessing the diffusion potential of an innovation were covered by the questionnaires. The questionnaire asked the respondents to estimate on a five-graded Likert scale whether they agreed or disagreed with a set of statements for each of the three diffusion of innovation attributes. For each attribute, questions pertinent for the study context were carefully selected. For example, the compatibility attribute was covered by questions regarding the functions of the CPOE system, e.g. the functions supporting all types of prescribing (oral, injections, inhalations and infusions). For the relative advantages attribute, relevant comparative questions were identified, e.g. whether the CPOE system was easier and faster to manage than paper document. For the complexity attribute, constrains that could limit the CPOE system use were asked for, e.g. whether the system lead to more adverse drug events and whether it required double documentation (on paper and in the CPOE system).

The questionnaires also asked for data on demographic characteristics of the study population and an overall assessment of the introduction of the CPOE system and each of the attributes relative advantages, complexity, and compatibility with values and needs.

The two questionnaires consisted of both identical and different items. For example, items asked the physicians about whether the CPOE system allowed them to be more efficient in clinical decision-making in ordering prescriptions, and the nurses about the system's capability to support medications in the context of home visits. Identical questions were asked to physicians and nurses about whether the system supported prescribing medicine by different routes (oral, injection, inhalation, and infusion) and about whether the system saved time and was faster and easier than the previous system.

To increase the likelihood that the questionnaires would serve their purpose [22], their face validity was assessed by gathering 6 professionals' opinions with backgrounds in health informatics, pharmacology, social medicine, economic information system, and statistics. After face validation of the questionnaires, we pilot tested them with 6 physicians and 3 nurses and allowed them to comment on the questionnaires as well. The questionnaires were revised according to their feedback.

### Study Population

The study population consisted of all physicians using the system and the nurses responsible for CPOE system at the clinics using the system in Östergötland county. 741 physicians and 200 nurses were identified as being eligible to be included in the study by the division in charge of the CPOE system introduction at the county council.

### Data Collection

The questionnaires were distributed in February 2009 through an online survey. The physicians and nurses were contacted by e-mail and asked to complete the questionnaires online. A first reminder was sent by e-mail in March. By early April, we had received 41 responses from the physicians and 186 responses from the nurses. To obtain more responses from the physicians, we tried to concentrate on those who work more with the CPOE system via distribution lists of physicians separated by clinic. We contacted the physicians again by sending the link for the survey to the identified e-mail lists, with a reminder after 2 weeks.

Of 200 surveys distributed to nurses, 186 were returned (overall response, 93.0%). Of 741 surveys to physicians, 211 were returned (overall response, 28.5%). However, 52 of the nurses' questionnaires and 35 of the physicians' questionnaires were excluded as incomplete. Thus, the total number of questionnaires included was 134 from 200 nurses (analyzed responses, 67.0%) and 176 from 741 physicians (23.8%).

### Data Analyses

After collecting data, we collapsed the Likert scale (quite agree, agree, neutral, quite disagree and disagree) into three grades (agree, neutral, and disagree) to facilitate the data analysis. A group-level index was also composed for each of the diffusion of innovation areas investigated. A personal agreement ratio was computed for each respondent, consisting of the proportion (0-100%) of the statements that the respondent agreed with. A group-level index was then computed as the mean of the personal agreement proportions.

Descriptive statistical methods were used to compute means and frequency distributions for the data set. Differences in respondent profile and level of agreement were tested for significance using chi-square test (and Fisher's Exact Test when necessary). Statistical significance was determined by P < 0.05. The statistical software, SPSS (Statistical Product and Services Solutions, version 16.0, SPSS Inc, Chicago, IL, USA) was used for analysis.

### Ethics statement

The study design used for the research reported in the article did not involve patient or laboratory data, but only investigates users' opinion about the CPOE system without experimentation. The study did thereby not require a formal ethical approval according to Swedish legislation. Participation in the study was both voluntary and anonymous. There was no way for us to identify the individual participants and thereby the participants are free to express their honest opinions about the system. Further, the study did not involve any sort of medical record review thereby protecting patient confidentiality.

## Results

The characteristics of the responding physicians and nurses are given in Table 1. With regard to level of training and occupation for the physicians, 6 (3.4%) were interns, 29 (16.5%) were residents, 23 (13.1%) were junior specialist physicians, 81 (46.0%) were senior consultant physicians, 31 (17.6%) were general practitioners, and 6 (3.4%) were other. Among nurses, 5 (5.7%) were district nurses or midwives with own practice, and the remaining 129 (96.3%) were nurses working in group practices (Table 1).

**Table 1:** Characteristics of the physicians and nurses in the final study population.

Characteristic	Physicians *n *= 176	Nurses *n *= 134
**Sex**		

Male	98 (55.7%	13 (9.7%)

Female	78 (44.3%)	121 (90.3%)

**Age groups**		

20-29 y	9 (5.1%)	3 (2.2%)

30-39 y	40 (22.9%)	34 (25.4%)

40-49 y	49 (28.%)	42 (31.3%)

50-59 y	53 (30.3%)	44 (32.8%)

> 60 y	24 (13.7%)	11 (8.2%)

**Workplace**		

Primary health care center	43 (24.4%)	9 (6.7%)

Hospital	133 (75.6%)	117 (87.3%)

Home care	0	8 (6.0%)

**County district**		

Central	102 (58.0%)	86 (64.2%)

Eastern	36 (20.5%)	26 (19.4%)

Western	38 (21.5%)	22 (16.4%)

**Time of CPOE system use**		

< 6 months	47 (26.7%)	43 (32.1%)

6-12 months	34 (19.3%)	47 (35.1%)

> 1 year	95 (54.0%)	44 (32.8%)

**Number of orders in a normal day**		

> 20	45 (25.6%)	51 (38.1%)

10-20	76 (43.2%)	27 (20.1%)

< 10	55 (31.2%)	56 (41.8%)

More nurses (56.7%) than physicians (31.3%) stated that the CPOE system introduction had worked well (good or very good) in their clinical setting (P < 0.001). There was no difference in the level of satisfaction with regard to workplace (P = 0.137), county district (P = 0.629), length of CPOE system use (P = 0.526) or number of orders prescribed in a normal day, the number of orders in a normal day refers to orders entered for physicians and orders managed for nurses (P = 0.210).

Similarly, more physicians (73.9%) than nurses (50.7%) reported that they found the system not adapted to their specific professional practice (*P *= < 0.001). There were no differences between workplaces (*P *= 0.865), county districts (*P *= 0.974) or individuals with different length of CPOE system use (*P *= 0.482) and number of orders prescribed in a normal day (*P *= 0.287). Also more physicians (25.0%) than nurses (13.4%) stated that they did want to return to the previous system (*P *= 0.041). There was no difference with regard to workplaces (*P *= 0.182), county district (*P *= 0.553), length of CPOE system use (*P *= 0.553) or number of orders prescribed in a normal day (*P *= 0.588).

**Figure 2:**
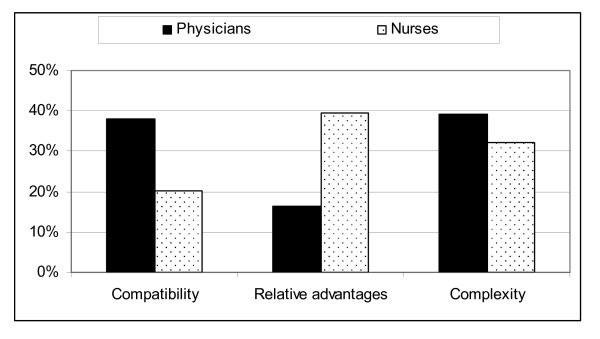
**Proportion of physicians and nurses who agreed with statements regarding the CPOE system's compatibility, relative advantages, and complexity of use**. A figure showing the physicians and nurses' agreement with statements regarding the CPOE system's compatibility, relative advantages, and complexity of use.

When comparing the composed index for the three attributions of the diffusion of innovation theory (Figure 2), we found that the received relative advantages of the CPOE system were estimated to be significantly (*P *< 0.001) higher among nurses (39.6%) than physicians (16.5%). Rather paradoxically, the physicians found the CPOE to be both more compatible with their professional values (*P *< 0.001) and more complex to use (*P *< 0.001) than nurses. Among physicians, the received relative advantages of the CPOE system (*P *< 0.575) as well as compatibility of the system (*P *< 0.150) displayed no significant differences between the workplaces. However, hospital physicians' agreement with that the CPOE was complex to use was significantly higher than among physicians who worked in primary healthcare (P < 001). Among the nurses, when comparing the composed index for the three attributes of the diffusion of innovation theory between workplaces, we found no significant difference for any of the attributes.

**Figure 3:**
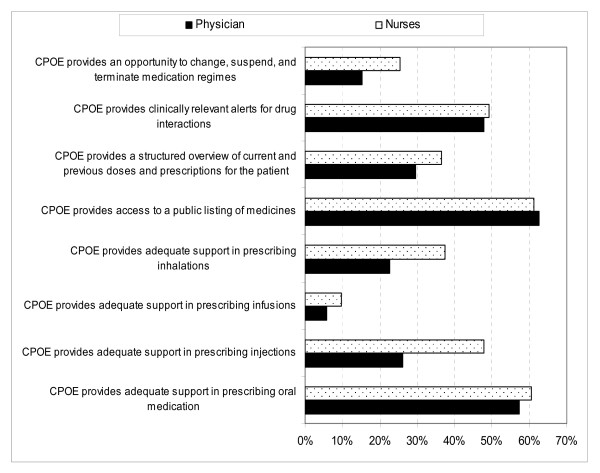
**Distribution of the respondents' agreement with statements about whether the CPOE system is compatible with professional values and needs**. A figure showing the physicians and nurses' agreement with statements about whether the CPOE system is compatible with professional values and needs.

A large share of the physicians and nurses agreed that the CPOE system provides access to a public list of medicines (62.5% and 61.2%, respectively), supplies adequate support in prescribing oral medicine (57.4% and 60.4%, respectively), and provides clinically relevant alerts for drug interactions (47.7% and 49.3%, respectively). However, only 5.7% of the physicians and 9.7% of nurses agreed that the system provides adequate support in prescribing medication by infusion, and only 15.3% of the physicians and 25.4% of the nurses agreed that the system provides an opportunity to create, change, suspend, and terminate medication regimens (Figure 3).

**Figure 4:**
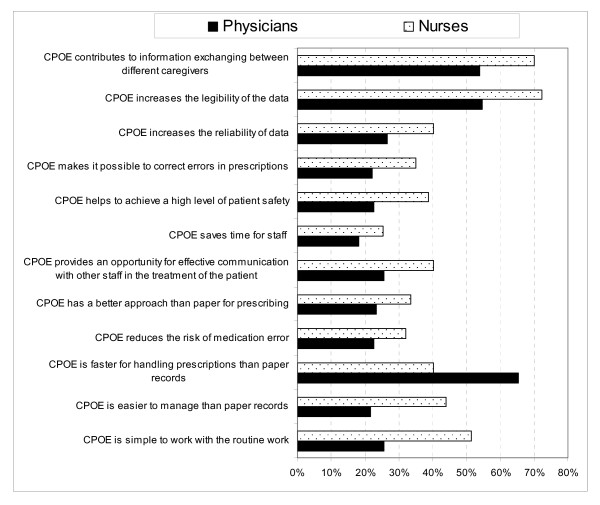
**Distribution of respondents' agreement with statements about the received relative advantages of the CPOE system**. A figure showing the physicians and nurses' agreement with statements about the received relative advantages of the CPOE system.

The respondents offered diverse opinions about the relative advantages of the CPOE system on work efficiency and patient safety (Figure 4). Most of the physicians (65.3%), but fewer nurses (40.3%), agreed that the system was faster to handle than the paper-based system. However, fewer physicians (54.5%) than nurses (72.4%) agreed that the system increased the legibility of prescriptions. In addition, 54% of physicians and 70.1% of the nurses agreed that the system contributed to better information exchange between different caregivers. A low percentage of the physicians (18.2%) and nurses (25.4%) agreed that the system saved time for staff. It is noteworthy that regarding patient safety, few of the respondents agreed that the system reduced the risk of medication error (22.7% of the physicians and 32.1% of the nurses) and that the system helped to achieve a high level of patient safety (22.7% of the physicians and 38.8% of the nurses).

**Figure 5:**
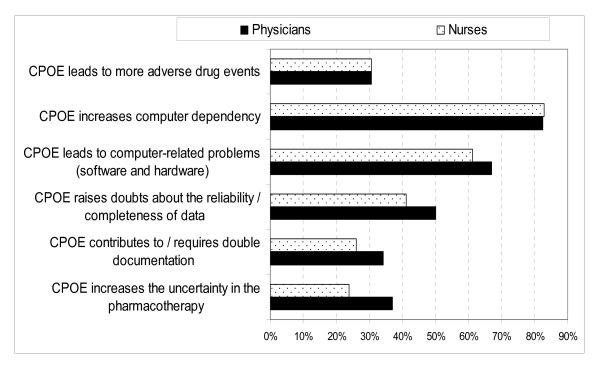
**Distribution of respondents' agreement with statements about complexity of the CPOE system**. A figure showing the physicians and nurses' agreement with statements about complexity of the CPOE system.

Most physicians (82.4%) and nurses (82.8%) agreed that the CPOE system increased computer dependency. In addition, 67% of the physicians and 61.2% of the nurses agreed that the system led to computer-related problems (software and hardware), which impacted on time use. The physicians (50%) and nurses (41%) agreed that the system raised doubts about reliability/completeness of data (Figure 5). Notably, only 30.7% of the physicians and 30.6% of the nurses agreed that the system introduction led to more adverse drug events.

## Discussion

We analyzed physicians and nurses' experiences related to the adoption of a CPOE system, structuring the analyses according to three attributes of diffusion of innovation theory, i.e. the relative advantage of the system, its compatibility with professional values and needs, and its complexity of use. Three-quarters of physicians and one-half of nurses found that the system was not adapted to their specific professional practice. Nurses were estimated to receive a higher relative advantage from having the system introduced in their work routines than physicians, while physicians found the system being more compatible with their professional values but more complex to use than nurses. Although disappointing, this pattern is not surprising in light of previous research and due to the fact that CPOE systems are mainly designed to support nurses in administering these decisions to patients, while supporting physicians' clinical decision-making.

The results indicate that an important reason behind the reluctance of physicians and nurses to use the CPOE system was that the system was not adapted to their work routines. When developing a clinical computer system that users interact with in their daily practice, consideration of the users' professional requirements must be at the core of the system implementation process [18,20]. In the study setting, the respondents seemed to be negative towards the CPOE system due to productivity losses, e.g. as consequences of human-computer interaction problems. Previous studies have shown that CPOE systems can increase productivity by making it possible to execute orders faster and easier than using paper technology [14,23,24]. However, the results of our study showed that most physicians and the nurses disagreed that the system saved time for them and was as easy to manage as paper documents. But although prescriptions may have taken more time per order using the CPOE system than paper, time can be saved during sequential tasks, for example, by being able to review the orders without having to use paper [25]. Such an interpretation is supported by the fact that a majority of the physicians and nurses in our study did not want to return to paper documents.

Moreover, patient safety has been identified as one of the most important advantages of CPOE systems, if not the most important. In Bates et al [3] and Ammenwerth et al. [26] studies, the safety effects have been reported to be mediated through two mechanisms, avoidance of mistakes (increased prescription legibility and possibility to correct misunderstandings) and support for evidence-based prescriptions.

Alertness is particularly important in light of the results of several previous studies that reported that CPOE systems led to a number of errors and adverse drug events [27-29]. In fact, about 30% of the physicians and nurses in our study indicated that the system could lead to more adverse drug events. As stated by Ash et al. [18], there are unintended consequences since not all outcomes can be foreseen such as error and security concerns.

Our results show that physicians and nurses agreed about increased prescription legibility. However, the results also show that most physicians and nurses disagreed that the CPOE system supported evidence-based pharmacological decision-making. In this regard, our results are consistent with previous studies [30]. One important reason for the disappointment with the decision support could be identified in our data, i.e. the insufficient compatibility between the system and clinical tasks. The system was intended to cover all kinds of prescriptions as well as to change medication regimens and provide clinically relevant alerts for drug interactions [21], failed to meet these requirements. More research is thus required on the clinical decision-making associated with prescriptions. If the system does not allow executing critical actions in the fulfillment of this category of tasks, the consequences can be fatal.

A CPOE system with integrated clinical decision support can be an advantage for the busy clinician who must combine and manage an increasing body of clinical knowledge. However, such support will not be optimal if clinicians begin to trust these systems without questioning the assistance [31]. Recent research on safety in human-machine interaction suggests that the presence of environmental cues reflecting hazards increases alertness among decision-makers and reduces the risk of mistakes [32]. From this perspective, it is positive that the system users had doubts about the reliability and completeness of the support provided by the system.

The present study has some important limitations. First, non-response leads to a smaller study group and possible loss of accuracy in the analyses. Despite the good response from the nurses, the non-responding physicians constituted more than half of the original population. It is not possible for us to know whether the respondents differed from the non-respondents in their attitudes toward the CPOE system, but non-responders did not differ from responders with regard to age, sex, and occupation. Second, only quantitative analyses were performed in this study. To analyze the individual and specific consequences and problems, qualitative data could have been more appropriate. A final limitation is that we did not formally validate the questionnaire items with regard to diffusion of innovation theory. The face validity of the separate questions was examined in several steps, as described in the Methods section.

## Conclusion

The risks of ineffective implementation and adoption of CPOE systems are high, as well as the risk for unintended consequences [12,26]. The importance of understanding the concerns of CPOE system users has been highlighted previously [33]. The diffusion of innovations theory with its attributes was found to be well suited for describing the organization-wide adoption of CPOE systems. According to the results, the qualifications for adoption as defined by the three attributes of diffusion of innovation theory were not satisfied in the study setting. Regarding the compatibility attribute of the CPOE system and the respondents concerns about the system adaption to their professional practice, it can be concluded that to decrease this concern, it is likely that performing needs assessments and supporting active physician and nurse involvement in the design and preparation will result in higher levels of satisfaction and use of CPOEs.

CPOE systems are introduced as a response to the present limitations in paper-based systems. In consequence, user expectations are often high on their relative advantages as well as on a low level of complexity. Building CPOE systems therefore requires designs that can provide rather important additional advantages compared to traditional means, e.g. by eliminating ambiguous handwriting, preventing prescription errors, increasing efficiency, producing cost savings, and ultimately improving patient safety and safety of clinical work [12,34]. In spite of the enormous investment in HISs such as CPOEs, however, the outcomes of many implementations have not met all the expectations [18,35,36]. This means that the decision-making process leading to the implementation and use of CPOE systems in healthcare has to be improved. As any change in health service settings usually faces resistance [37-39], we emphasize that CPOE system designers and healthcare decision-makers should continually collect users' feedback about the systems, while not forgetting that it also is necessary to inform the users about the potential benefits involved [33].

## Competing interests

The authors declare that they have no competing interests.

## Authors' contributions

BR was the responsible for the study design, data analysis, interpretation of results, and drafting the manuscript. TT contributed to the design of the study, data analysis, and revision of the manuscript. VV contributed to the design of the study and revision of the manuscript. SU and MS contributed to the design of the study, gathering the data, and revision of the manuscript. All authors read and approved the final manuscript.

## Pre-publication history

The pre-publication history for this paper can be accessed here:

http://www.biomedcentral.com/1472-6947/9/52/prepub

## Supplementary Material

Additional file 1 **An online survey questionnaire for physicians**. The questionnaire is part of a research study focused on analysis of the effects achieved through the use of computerized provider order entry (CPOE) in health care. Click here for file

Additional file 2 **An online survey questionnaire for nurses**. The questionnaire is part of a research study focused on analysis of the effects achieved through the use of computerized provider order entry (CPOE) in health care. Click here for file

## References

[B1] Kaushal R Shojania KG Bates DW Effects of computerized physician order entry and clinical decision support systems on medication safety: a systematic review Arch Intern Med 2003 163 12 1409 1416 10.1001/archinte.163.12.1409 12824090

[B2] Kohn LT Corrigan JM Donaldson MS editors To err is human: building a safer health system 1999 Washington DC: Institute of Medicine report. National Academies Press 25077248

[B3] Bates DW Kuperman G Teich JM Computerized physician order entry and quality of care Qual Manag Health Care 1994 2 4 18 27 10172133

[B4] Eslami S de Keizer NF Abu-Hanna A The impact of computerized physician medication order entry in hospitalized patients--a systematic review Int J Med Inform 2008 77 6 365 376 10.1016/j.ijmedinf.2007.10.001 18023611

[B5] Rahimi B Vimarlund V Methods to Evaluate Health information Systems in Healthcare Settings: A Literature Review J Med Syst 2007 31 5 397 432 10.1007/s10916-007-9082-z 17918694

[B6] Mekhjian HS Kumar RR Kuehn L Bentley TD Teater P Thomas A Payne B Ahmad A Immediate benefits realized following implementation of physician order entry at an academic medical center J Am Med Inform Assoc 2002 9 5 529 539 10.1197/jamia.M1038 12223505 PMC346640

[B7] Campbell EM Sittig DF Ash JS Guappone KP Dykstra RH Types of unintended consequences related to computerized provider order entry J Am Med Inform Assoc 2006 13 5 547 556 10.1197/jamia.M2042 16799128 PMC1561794

[B8] Ash JS Sittig DF Poon EG Guappone K Campbell E Dykstra RH The extent and importance of unintended consequences related to computerized provider order entry J Am Med Inform Assoc 2007 14 4 415 423 10.1197/jamia.M2373 17460127 PMC2244906

[B9] Doolan DF Bates DW Computerized physician order entry systems in hospitals: mandates and incentives Health Aff (Millwood) 2002 21 4 180 188 10.1377/hlthaff.21.4.180 12117128

[B10] Kaushal R Bates DW Information technology and medication safety: what is the benefit? Qual Saf Health Care 2002 11 3 261 265 10.1136/qhc.11.3.261 12486992 PMC1743646

[B11] Niazkhani Z Pirnejad H Berg M Aarts J The impact of computerized provider order entry systems on inpatient clinical workflow: a literature review J Am Med Inform Assoc 2009 16 4 539 549 10.1197/jamia.M2419 19390113 PMC2705258

[B12] Georgiou A Ampt A Creswick N Westbrook JI Braithwaite J Computerized Provider Order Entry--what are health professionals concerned about? A qualitative study in an Australian hospital Int J Med Inform 2009 78 1 60 70 10.1016/j.ijmedinf.2008.09.007 19010728

[B13] Lorenzi NM Kouroubali A Detmer DE Bloomrosen M How to successfully select and implement electronic health records (EHR) in small ambulatory practice settings BMC Med Inform Decis Mak 2009 9 15 10.1186/1472-6947-9-15 19236705 PMC2662829

[B14] Pirnejad H Niazkhani Z Sijs H van der Berg M Bal RA Evaluation of the Impact of a CPOE System on Nurse-physician Communication - A Mixed Method Study Methods Inf Med 2009 48 4 350 360 19448880 10.3414/ME0572

[B15] Lindenauer PK Ling D Pekow PS Crawford A Naglieri-Prescod D Hoople N Fitzgerald J Benjamin EM Physician characteristics, attitudes, and use of computerized order entry J Hosp Med 2006 1 4 221 230 10.1002/jhm.106 17219503

[B16] Aarts J Koppel R Implementation of computerized physician order entry in seven countries Health Aff (Millwood) 2009 28 2 404 414 10.1377/hlthaff.28.2.404 19275996

[B17] Rogers EM Diffusion of innovations 2003 5 New York: Free press

[B18] Ash JS Sittig DF Dykstra RH Guappone K Carpenter JD Seshadri V Categorizing the unintended sociotechnical consequences of computerized provider order entry Int J Med Inform 2007 76 Suppl 1 S21 7 10.1016/j.ijmedinf.2006.05.017 16793330

[B19] Ford EW McAlearney AS Phillips MT Menachemi N Rudolph B Predicting computerized physician order entry system adoption in US hospitals: can the federal mandate be met? Int J Med Inform 2008 77 8 539 545 10.1016/j.ijmedinf.2007.10.009 18053762

[B20] Berwick DM Disseminating innovations in health care JAMA 2003 289 15 1969 1975 10.1001/jama.289.15.1969 12697800

[B21] Cambio Healthcare System COSMIC Order Management - It couldn't be any simpler 2009 http://www.cambio.se/document/en-us/Clinical_Eng.pdf Accessed June 5, 2009

[B22] Brender J Handbook of evaluation methods for health informatics 2006 Amsterdam: Elsevier Academic Press

[B23] Classen DC Avery AJ Bates DW Evaluation and certification of computerized provider order entry systems J Am Med Inform Assoc 2007 14 1 48 55 10.1197/jamia.M2248 17077453 PMC2215075

[B24] Zhan C Hicks RW Blanchette CM Keyes MA Cousins DD Potential benefits and problems with computerized prescriber order entry: analysis of a voluntary medication error-reporting database Am J Health Syst Pharm 2006 63 4 353 358 10.2146/ajhp050379 16452521

[B25] Ash JS Bates DW Factors and forces affecting EHR system adoption: report of a 2004 ACMI discussion J Am Med Inform Assoc 2005 12 1 8 12 10.1197/jamia.M1684 15492027 PMC543830

[B26] Ammenwerth E Schnell-Inderst P Machan C Siebert U The Effect of Electronic Prescribing on Medication Errors and Adverse Drug Events: A Systematic Review J Am Med Inform Assoc 2008 15 5 585 600 10.1197/jamia.M2667 18579832 PMC2528040

[B27] Han YY Carcillo JA Venkataraman ST Clark RS Watson RS Nguyen TC Bayir H Orr RA Unexpected increased mortality after implementation of a commercially sold computerized physician order entry system Pediatrics 2005 116 6 1506 1512 10.1542/peds.2005-1287 16322178

[B28] Nebeker JR Hoffman JM Weir CR Bennett CL Hurdle JF High rates of adverse drug events in a highly computerized hospital Arch Intern Med 2005 165 10 1111 1116 10.1001/archinte.165.10.1111 15911723

[B29] Weiner M Gress T Thiemann DR Jenckes M Reel SL Mandell SF Bass EB Contrasting views of physicians and nurses about an inpatient computer-based provider order-entry system J Am Med Inform Assoc 1999 6 3 234 244 10332656 10.1136/jamia.1999.0060234 PMC61363

[B30] Ammenwerth E Mansmann U Iller C Eichstadter R Factors affecting and affected by user acceptance of computer-based nursing documentation: results of a two-year study J Am Med Inform Assoc 2003 10 1 69 84 10.1197/jamia.M1118 12509358 PMC150360

[B31] Campbell EM Sittig DF Guappone KP Dykstra RH Ash JS Overdependence on technology: an unintended adverse consequence of computerized provider order entry AMIA Annu Symp Proc 2007 94 98 18693805 PMC2710605

[B32] Norman DA The design of future things 2007 New York, NY: Basic Books

[B33] Sittig DF Krall M Kaalaas-Sittig J Ash JS Emotional aspects of computer-based provider order entry: a qualitative study J Am Med Inform Assoc 2005 12 5 561 567 10.1197/jamia.M1711 15905478 PMC1205605

[B34] Ammenwerth E Talmon J Ash JS Bates DW Beuscart-Zephir MC Duhamel A Elkin PL Gardner RM Geissbuhler A Impact of CPOE on mortality rates--contradictory findings, important messages Methods Inf Med 2006 45 6 586 593 17149499

[B35] Fullerton C Aponte P Hopkins R Bragg D Ballard DJ Lessons learned from pilot site implementation of an ambulatory electronic health record Proc(Bayl Univ Med Cent) 2006 19 4 303 310 17106488 10.1080/08998280.2006.11928188 PMC1618740

[B36] Meijden MJ Van Der Tange HJ Troost J Hasman A Determinants of success of inpatient clinical information systems: a literature review J Am Med Inform Assoc 2003 10 3 235 243 10.1197/jamia.M1094 12626373 PMC342046

[B37] Grol R Personal paper. Beliefs and evidence in changing clinical practice BMJ 1997 315 7105 418 421 9277610 10.1136/bmj.315.7105.418 PMC2127297

[B38] Rahimi B Moberg A Timpka T Vimarlund V Implementing an integrated computerized patient record system: Towards an evidence-based information system implementation practice in healthcare AMIA Annu Symp Proc 2008 616 620 18999062 PMC2655989

[B39] Rahimi B Vimarlund V Timpka T Health Information System Implementation: A Qualitative Meta-analysis J Med Syst 2009 33 5 359 368 10.1007/s10916-008-9198-9 19827262

