# Supplementary material for: Organization-wide adoption of computerized provider order entry systems: a study based on diffusion of innovations theory
Source: BMC Med Inform Decis Mak. 2009 Dec 31;9:52. doi: 10.1186/1472-6947-9-52 (PMC2809050; doi:10.1186/1472-6947-9-52)
Supplement: Additional file 1 — An online survey questionnaire for physicians. The questionnaire is part of a research study focused on analysis of the effects achieved through the use of computerized provider order entry (CPOE) in health care. [file 1472-6947-9-52-S1.DOC]

| 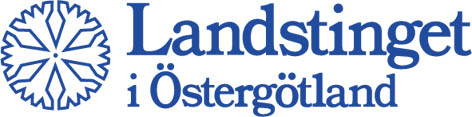 | 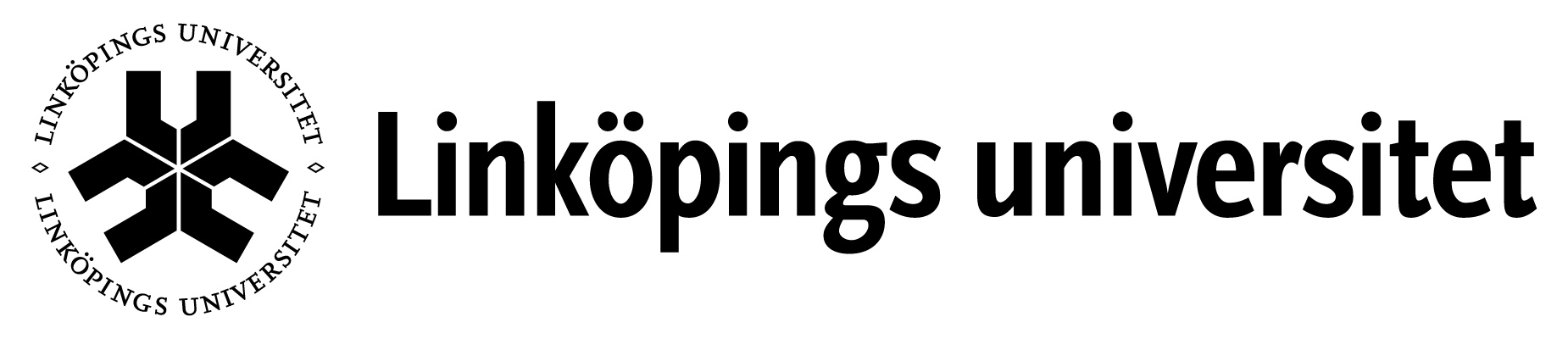 |
| --- | --- |
| Questionnaire for physicians | |

# This questionnaire is sent to you in your role as a clinical physician. You are currently using a CPOE at your workplace. The questionnaire is part of a research study focused on analysis of the effects achieved through the use of CPOE in health care. The questionnaire includes questions about your professional background, how much you come into contact with the CPOE system, how effective you perceive that it is, and what effect its introduction may have caused. A high response rate increases the validity of the study. Answering the survey is estimated to take between 15 to 25 minutes.

**Voluntary participation:** It is voluntary to participate in the study and you can refrain from participation at any time without having to explain why. There is no reimbursement for participation.

**Confidentiality:** We will handle the data anonymously and with confidentiality. Access to primary reported material will be restricted to the research group (your employer will not have access to your answers). It will not be possible to detect individuals when reading reports from the study.

Please do not hesitate to ask any questions.

Mikael Svensson,
Apotekare, Samordnare,
Landstinget i Östergötland
Tfn: 013 227361
mikael.svensson@lio.se

Bahlol Rahimi
Doktorand
Institutionen för datavetenskap
Linköpings universitetet
bahra@ida.liu.se

# Toomas Timpka

# Professor

Section of Social Medicine and Public Health Sciences,

Linköping University

tti@ida.liu.se

**A) General questions**

1. **What is your current position:**

□ Intern

□ Resident physician

□ Consultant physician

□ Senior consultant physician

□ Primary care physician/general practitioner

□ Other, namely _____________

1. **Age:** …………………years old
2. **Sex:** □ Male □ Female
3. **Workplace**: □PHC □Hospital
4. **Department name:** ……………………………………………………
5. **County district:**

□ Central district

□ East district

□ West district

1. **Type of work:** □ Fulltime □ part-time.......%
2. **Experience at your present position:**

□< 1 year

□1-3 years

□3-5 years

□> 5 years

1. **Experience from use of CPOE system?**

□< 1 month

□ 2-6 months

□6-12 months

□ > 1 year

1. **Entered orders in the CPOE system in a normal day**

□ Very many ( >20)

□ Many (10-20)

□ Few ( <10)

□ None

1. **Received training on CPOE system usage?**

□Yes

□ No -if yes, answer questions 12 and 13

1. **How long was the training??** ........................... hour(s)
2. **How has the training of the CPOE system been?**

□ Good

□ Rather good

□ Bad

1. **Opportunity to practice during working hours on your own workplace to learn the CPOE system**

□Yes

□ No - if yes, answer questions 15 and 16

1. **How long (non-booked hours) did you get the opportunity to practice? .................. hour(s)**
2. **How has the self practicing of the CPOE system been?**

□ Good

□ Rather good

□ Bad

**B) Efficiency**

**17. Indicate to what extent you agree with the following statements:
The CPOE system provides adequate support for**:

|  | **Agree** | **Partly agree** | **Neutral** | **Party disagree** | **disagree** |
| --- | --- | --- | --- | --- | --- |
| - Oral medications |  |  |  |  |  |
| - Injections |  |  |  |  |  |
| - Infusions |  |  |  |  |  |
| - Inhalations |  |  |  |  |  |

**18. Indicate to what extent you agree with the following statements:
The CPOE system:**

|  | **Agree** | **Partly agree** | **Neutral** | **Party disagree** | **disagree** |
| --- | --- | --- | --- | --- | --- |
| - 1. provides access to a public listing of medicines |  |  |  |  |  |
| - 2. provides a structured overview of current and previous dosages and prescriptions for the patient |  |  |  |  |  |
| - 3. provides clinically relevant alerts for drug interactions |  |  |  |  |  |
| 4. is easy to use with in the routine work |  |  |  |  |  |
| 5. provides an opportunity to change, suspend, and terminate medication regimes |  |  |  |  |  |
| 6. is easier to manage than paper records |  |  |  |  |  |
| 7. is faster for handling the prescription than the paper record |  |  |  |  |  |
| 8. reduces the risk of prescribing error |  |  |  |  |  |
| 9. has a better approach than paper for prescribing |  |  |  |  |  |
| 10. allows more efficient decision-making when you want to prescribe drugs |  |  |  |  |  |
| 11. provides an opportunity for effective communication with other staff in the treatment of the patient |  |  |  |  |  |
| 12. saves time for staff |  |  |  |  |  |

1. **How has the CPOE system function so far worked in your practice?**

□Very good

□Good

□No difference

□ Bad

□Very bad

1. **Has the system use over the last ten days when you used the system, enabled you to prevent medication error before the error reached the client / patient?**

□Yes

□No

1. **If yes, how many such errors have you discovered?**

□1

□1-3

□4-10

□>10

1. **Have there been times over the last ten days when you use the system, where the use of the system caused a medication error?**

□Yes

□No

1. **If yes, how many such incidents encountered at?**

□1

□1-3

□4-10

□>10

**C) Outcome of the CPOE system use**

1. **Indicate to what extent you agree with the following statements:
   The CPOE system:**

|  | **Agree** | **Partly agree** | **Neutral** | **Party disagree** | **disagree** |
| --- | --- | --- | --- | --- | --- |
| - 1. helps to achieve a high level of patient safety |  |  |  |  |  |
| - 2. makes it possible to correct errors in prescriptions |  |  |  |  |  |
| - 3. provides clinical decision-making support when the physician wants to prescribe medicines |  |  |  |  |  |
| 4. increases the reliability of data |  |  |  |  |  |
| 5. increases the legibility of the data |  |  |  |  |  |
| 6. contributes to information exchanging between different caregiver |  |  |  |  |  |
| 7. increases un-safety in the pharmacotherapy |  |  |  |  |  |
| 8. contributes to / requires double documentation (on paper and in the CPOE) |  |  |  |  |  |
| 9. cause doubts about reliability / completeness of data |  |  |  |  |  |
| 10. leads to computer-related problems (software and hardware), which impacts on time |  |  |  |  |  |
| 11. increasing computer dependency |  |  |  |  |  |
| 12. leads to more adverse drug events |  |  |  |  |  |

1. **Would you like to completely go back to the previous system?**

□Yes □No □Do not know

If yes, why not?____________________________________________________

1. **Do you think the CPOE is adapted to your professional needs?**

□Yes □No

If no, why:_______________________________________________________

1. **What problems do you think has been solved by the introduction of CPOE system?**
2. **What new problems do you have emerged since the introduction of CPOE system?**
