# Supplementary material for: Organization-wide adoption of computerized provider order entry systems: a study based on diffusion of innovations theory
Source: BMC Med Inform Decis Mak. 2009 Dec 31;9:52. doi: 10.1186/1472-6947-9-52 (PMC2809050; doi:10.1186/1472-6947-9-52)
Supplement: Additional file 2 — An online survey questionnaire for nurses. The questionnaire is part of a research study focused on analysis of the effects achieved through the use of computerized provider order entry (CPOE) in health care. [file 1472-6947-9-52-S2.DOC]

| 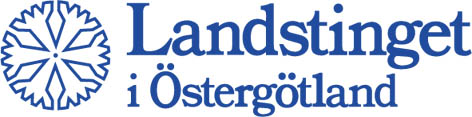 | 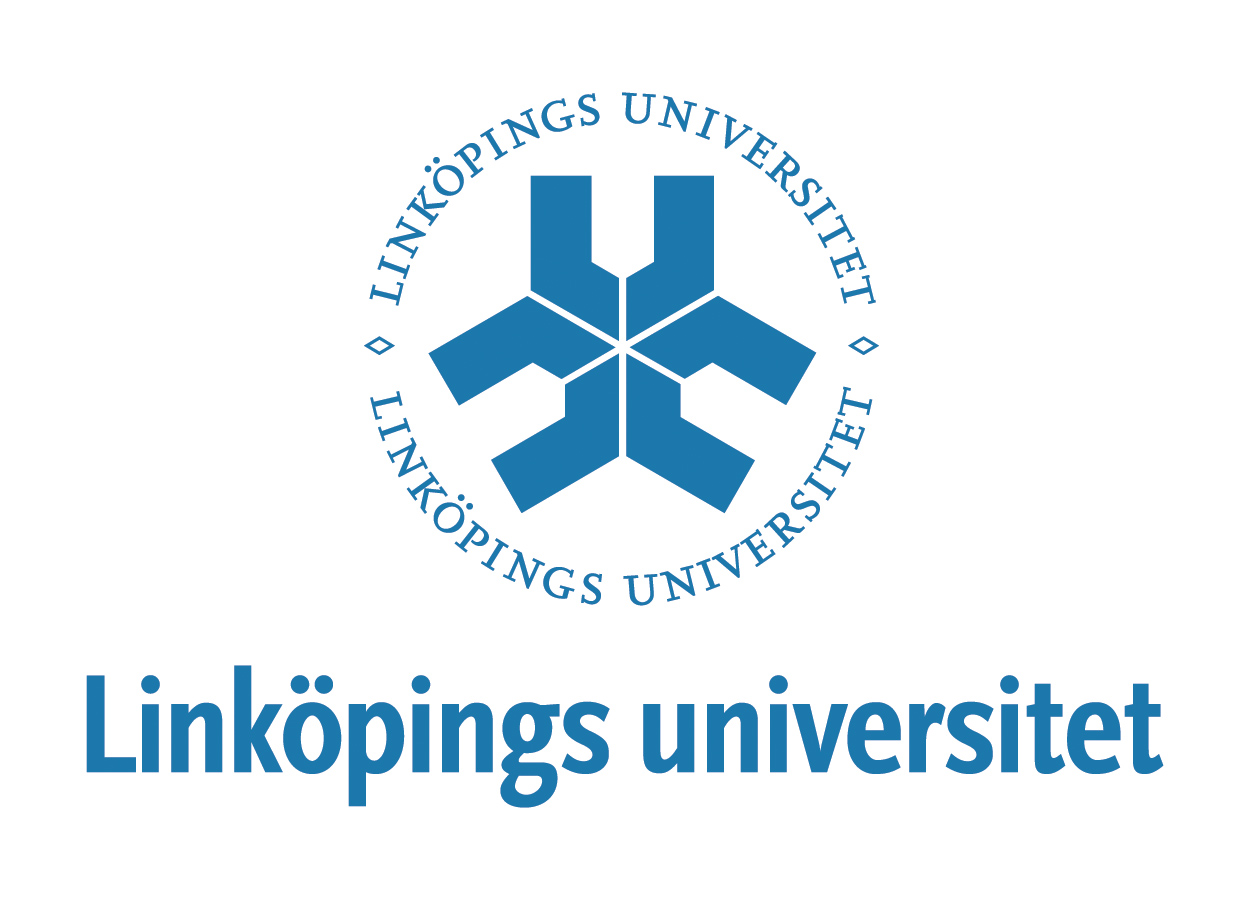 |
| --- | --- |
| Questionnaire for nurses | |

# You receive this questionnaire in your professional role as a nurse. You currently use a CPOE at your workplace. The questionnaire is part of a research study focused on analysis of the effects achieved through the introduction of CPOE in health care.

# This questionnaire includes questions about your professional background, how much you come into contact with the CPOE system, how effective you perceive that it is, and what effects its introduction may have caused. A high response rate increases the validity of the study. Answering the survey is estimated to take between 15 to 25 minutes.

**Voluntary participation:** It is voluntary to participate in the study and you can refrain from participation at any time without having to explain why. There is no reimbursement for participation.

**Confidentiality:** We will handle the data anonymously and with confidentiality. Access to primary reported material is restricted to the research group (your employer will not have access to your answers). Readers of reports from the study will not be able to identify individual responses.

Please do not hesitate to ask any questions.

# Bahlol Rahimi Srinivas Uppugunduri

# Doktoral student Pharmacist, docent

# Institutionen för datavetenskap

# Linköpings universitet Landstinget i Östergötland

# [**bahra@ida.liu.se**](mailto:bahra@ida.liu.se) Tfn: 013- 22 73 07

# [srinivas.uppugunduri@lio.se](mailto:srinivas.uppugunduri@lio.se)

# Mikael Svensson,

# Pharmacist,

# Tfn 013/227361

# e-post: mikael.svensson@lio.se

**A) General questions**

1. **What is your current position:**

□ Nurse

□ District nurses

□ Midwife

1. **Age:** …………………years old
2. **Sex:** □ Male □ Female
3. **Workplace**:

□Homecare

□PHC

□Hospital

1. **Department name:** ……………………………………………………
2. **County district:**

□ Central district

□ East district

□ West district

1. **Type of work:** □ Fulltime □ part-time.......%
2. **Experience from nursing practice:**

□< 1 year

□1-3 years

□3-5 years

□> 5 years

1. **Experience from use of CPOE system?**

□< 1 month

□ 2-6 months

□6-12 months

□ > 1 year

1. **Entered orders in the CPOE system in a normal day**

□ Very many ( >20)

□ Many (10-20)

□ Few ( <10)

□ None

1. **Received training on CPOE system usage?**

□Yes

□ No -if yes, answer questions 12 and 13

1. **How long was the training??** ........................... hour(s)
2. **How has the training of the CPOE system been?**

□ Good

□ Rather good

□ Bad

1. **Opportunity to practice during working hours on your own workplace to learn the CPOE system**

□Yes

□ No - if yes, answer questions 15 and 16

1. **How long (non-booked hours for scheduled training) did you get the opportunity to practice? .................. hour(s)**
2. **How has the self practicing of the CPOE system been?**

□ Good

□ Rather good

□ Bad

**B) Efficiency**

**17. Indicate to what extent you agree with the following statements:
The CPOE system provides adequate support for:**

|  | **Agree** | **Partly agree** | **Neutral** | **Party disagree** | **disagree** |
| --- | --- | --- | --- | --- | --- |
| - Oral medications |  |  |  |  |  |
| - Injections |  |  |  |  |  |
| - Infusions |  |  |  |  |  |
| - Inhalations |  |  |  |  |  |
| - Medication in connection with home visits |  |  |  |  |  |

**18. Indicate to what extent you agree with the following statements:
The CPOE system provides adequate support for:**

|  | **Agree** | **Partly agree** | **Neutral** | **Party disagree** | **disagree** |
| --- | --- | --- | --- | --- | --- |
| - change medication regimes |  |  |  |  |  |
| - suspend medication regimes |  |  |  |  |  |
| - terminate medication regimes |  |  |  |  |  |

**19. Indicate to what extent you agree with the following statements:**

| **The CPOE system** | **Agree** | **Partly agree** | **Neutral** | **Party disagree** | **disagree** |
| --- | --- | --- | --- | --- | --- |
| 1. provides access to a public listing of medicines |  |  |  |  |  |
| 1. provides a structured overview of current and previous dosages and prescriptions for the patient |  |  |  |  |  |
| 1. provides clinically relevant alerts for drug interactions |  |  |  |  |  |
| 1. is easy to work with in routine work |  |  |  |  |  |
| 1. is easier to manage than paper records |  |  |  |  |  |
| 1. is faster to handle for prescriptions than in the paper record |  |  |  |  |  |
| 1. reduces the risk of prescribing error |  |  |  |  |  |
| 1. has a better approach than paper for prescriptions |  |  |  |  |  |
| 1. provides an opportunity for effective communication with other staff in the treatment of the patient |  |  |  |  |  |
| 1. saves time for staff |  |  |  |  |  |

1. **How has the CPOE system function so far worked in your business?**

□Very good

□Good

□No differences

□ Bad

□Very bad

1. **Has the system use over the last ten days when you used the system, enabled you to prevent medication error before the error reached the client / patient?**

□Yes

□No

1. **If yes, how many such errors have you discovered?**

□1

□1-3

□4-10

□>10

1. **Have there been times over the last ten days when you use the system, where the use of the system caused a medication error?**

□Yes

□No

1. **If yes, how many such incidents have you encountered?**

□1

□1-3

□4-10

□>10

**C) Outcome of the CPOE system use**

1. **Indicate to what extent you agree with the following statements:
   The CPOE system:**

|  | **Agree** | **Partly agree** | **Neutral** | **Party disagree** | **disagree** |
| --- | --- | --- | --- | --- | --- |
| - 1. helps to achieve a high level of patient safety |  |  |  |  |  |
| - 2. makes it possible to correct errors in prescriptions |  |  |  |  |  |
| 3. increases the reliability of data |  |  |  |  |  |
| 4. increases the legibility of the data |  |  |  |  |  |
| 5. contributes to information exchange between different caregiver |  |  |  |  |  |
| 6. increases the un-safety in the pharmacotherapy |  |  |  |  |  |
| 7. contributes to / requires double documentation (both on paper and in the CPOE) |  |  |  |  |  |
| 8. causes doubts about reliability / completeness of data |  |  |  |  |  |
| 9. leads to computer-related problems (software and hardware), which impact on time |  |  |  |  |  |
| 10. increasing computer dependency |  |  |  |  |  |
| 11. leads to more adverse drug events |  |  |  |  |  |

1. **Would you like to completely go back to the previous system?**

□Yes □No □Do not know

If yes, why not?____________________________________________________

1. **Do you think the CPOE is adapted to your professional needs?**

□Yes □No

If no, why:_______________________________________________________

1. **What problems do you think have been solved by the introduction of CPOE system?**
2. **What new problems have emerged since the introduction of CPOE system?**
